# Supplementary material for: Towards blood flow in the virtual human: efficient self-coupling of HemeLB
Source: Interface Focus. 2020 Dec 11;11(1):20190119. doi: 10.1098/rsfs.2019.0119 (PMC7739917; doi:10.1098/rsfs.2019.0119)
Supplement: Supplementary Material: 1- MPI Function Details; 2 - Load Balancing Investigations [file rsfs20190119supp1.pdf]

Towards Blood Flow in the Virtual Human: Efficient Self-Coupling of HemeLB

McCullough, J.W.S.[1], Richardson, R.A.[1], Patronis, A.[1,2], Halver, R.[2], Marshall, R.[3], Ruefenacht, M.[3], Wylie, B.J.N.[2], Odaker, T.[4], Wiedemann, M.[4], Lloyd, B.[5], Neufeld, E.[5], Sutmann, G.[2,6], Skjellum, A.[3], Kranzlmüller, D.[4], and Coveney, P.V.[1,7]

[1] *Centre for Computational Science, Department of Chemistry, University College London, London, UK*
[2] *Jülich Supercomputing Centre, Forschungszentrum Jülich, Jülich, Germany*
[3] *SimCenter, University of Tennessee at Chattanooga, Chattanooga, TN, USA*
[4] *Leibniz Supercomputing Centre, Leibniz-Rechenzentrum (LRZ), Garching, Germany*
[5] *Foundation for Research on Information Technologies in Society (IT'IS), Zurich, Switzerland*
[6] *ICAMS, Ruhr-University Bochum, Bochum, Germany*
[7] *Informatics Institute, University of Amsterdam, Amsterdam, Netherlands*

Supplementary material for the article appearing in the theme issue of *Interface Focus* 'Computational Biomedicine Part 2: Organs and Systems'

1 Supplementary Material - MPI Function Details

The BigCount problem refers to a limitation in the MPI standard that restricts the number of data items in certain function calls to $2^{32} - 1$. The example below shows a simple use case, where a filename `infile` is opened and read as a series of integers into a buffer called `ibuff` having a number of elements equal to `count`. The value of `count` is based on the size of the input file. Though `count` is presumably safe on a native system, it is not supported by the current MPI standard for use as an argument to `MPI_File_read`. Thus, the call to `MPI_File_read` exhibits unexpected behaviour if the file size is sufficiently large to cause overflow, and in such cases, the value for the last element in `ibuff` is undefined.

Listing 1: User level C code attempting to read a large file under the current MPI standard.

```
MPI_File fh;
MPI_Status status;
MPI_Info info = MPI_INFO_NULL;
MPI_Count count = fs::file_size(infile) / sizeof(int);

int flags = (MPI_MODE_RDONLY);
```

```
int *ibuff = (int *)malloc((sizeof(int) * count));

MPI_File_open(MPI_COMM_WORLD, infile.c_str(), flags, info, &fh);
MPI_File_read(fh, &(ibuff[0]), count, MPI_INT, &status);
MPI_File_close(&fh);

// ibuff[count - 1] contains ???
```

HemeLB can take advantage of exascale today, but is hindered by limitations in the IO component of MPI. At scale, HemeLB has the opportunity to utilize a large number of data items that exceed the 32-bit limit when calling `MPI_File_read`. To work around this limitation, the data is broken into 32-bit safe segments and read in sequence. A generalized example of the procedure is shown below.

Listing 2: User level C++ code attempting to read a large file in separate segments when necessary (assume equal segment sizes).

```
void read_x(MPI_File fh, void *buf, MPI_Count count,
        MPI_Datatype datatype, MPI_Status *status) {

  char* cb = (char*)buf;
  MPI_Count
    segment_id = 0,
    limit = READ_X_MAX_BUFFER;

  if (count < limit) {
    // count can safely be cast to int, call regular File_read
    MPI_File_read(fh, buf, (int)count, datatype, status) ;
  } else {

    MPI_Count
      curr = 0,
      next = ((segment_id + 1) * limit),
      nsegments = (count / limit);

    while (segment_id < nsegments) {
      MPI_Count b = segment_id * limit;
      MPI_File_read_at (fh,curr, &cb[b], limit, datatype, status);

      segment_id++;
```

```
        curr = next;
        next += limit;
      }
    }
}
```

2 Supplementary Material - Load Balancing Investigations

Here we provide further detail regarding the load balancing techniques native to HemeLB and implemented via the ALL library. We then present initial results based on the performance of these techniques in distributing the workload of the circle of Willis geometry on SuperMUC-NG.

2.1 Load balancing with native HemeLB method

The original load balancing approach uses a successive workload assignment to processes. The workload characterisation is based on compute blocks containing 8^3 lattice sites. Each block is weighted by the sum of the relative computational effort of its constituent sites. When assigning work to P cores, blocks are consecutively imported on processor domains until the local work contribution, W_i, reaches W_t/P ($\pm$ one block), where W_t is the total work in the system. Concave geometrical regions in the system may force the algorithm to connect non-local regions, which results in an increased surface-to-volume ratio for several domains and is unfavourable for communication overhead.

2.2 Load balancing with histogram method

The selected load balancing scheme, which is adopted from the ALL library, uses orthogonal recursive bisection (ORB) combined with a histogram method. Here we have chosen a staggered mesh method, which is a special case of ORB and which reduces the number of steps in the construction phase compared with general ORB methods. Defining a subdivision of $P = \mathsf{P}_x \times \mathsf{P}_y \times \mathsf{P}_z$, a cumulative work distribution function is computed in a first step via a histogram method in one cartesian direction (e.g. the z-direction). Knowing the normalised histogram, $H_W(z)$, it can be split into P_z

slabs, containing similar work via partitioning the cumulative work-function,

$$W(z) = W_t \sum_{i}^{z_n \le z} H_W(z_i)\, \delta z \tag{1}$$

where δz is the histogram resolution. Accordingly, the width of the slabs can be computed via

$$\Delta z_i = W^{-1}((i+1)\, W_t/P) - W^{-1}(i\, W_t/P) \tag{2}$$

After the first step, P_z slabs exist, which are subdivided further. In each slab a communicator is created which assembles information from all cores having particles stored within this slab. Each slab is then further subdivided in, e.g. the y-direction, creating in each slab P_y columns as a result of a histogram partitioning in y-direction. The last step then consists in generating histograms along each column along the remaining x-direction and partitioning it accordingly into P_x boxes. This procedure then results in total in $P = P_x \times P_y \times P_z$ boxes. Due to the procedure of construction neighboured domains are, in general, staggered in the $y-$ and $z-$direction and the number of neighbours is not pre-defined. Since the histograms are constructed according to the work-weights of individual blocks processes have a very similar workload. Performance may still vary, since effects like communication overhead or memory access are not considered in the construction. Consideration of these effects is a matter of ongoing research.

2.3 Preliminary results

Figures 1 and 2 compare the total block weight on each process obtained from the original approach and from ALL. It is found that ALL is able to distribute the weights more homogeneously between all processes than the original approach, which shows a tendency to underweight the processors with highest core count. As a measure for positive and negative deviations from the optimal value, the average block weight is taken as a confidence interval (dashed red lines). The fact that weight fluctuations, obtained from the ALL library fall into this confidence interval means, that ALL is, in principle, able to find the optimal distribution of weights. Fluctuations occur due to the discrete nature of the blocks, i.e., it may happen that a dividing surface between processes cuts the total weight into two equal portions plus/minus one block weight. Since block weights are not constant, a distribution of weights around the average weight $\langle W \rangle = \sum_{i=0}^{P-1} w_i/P$ is obtained.

To compare the efficiency, two measures are considered:

$$\omega = \frac{\langle W \rangle}{W_{max}} \tag{3}$$

$$\eta = \frac{W_{max} - W_{min}}{W_{max} + W_{min}} \tag{4}$$

If we assume that an ideal distribution would result in a work distribution within the range $\langle W \rangle \pm \langle w_b \rangle$, then we can consider the upper limit η_{id}^{+} as

$$\eta_{id}^{+} = \frac{\langle W \rangle + \langle w_b \rangle - (\langle W \rangle - \langle w_b \rangle)}{\langle W \rangle + \langle w_b \rangle + (\langle W \rangle - \langle w_b \rangle)} = \frac{\langle w_b \rangle}{\langle W \rangle} \tag{5}$$

For ω we can define a reasonable lower and upper boundary as

$$\omega_{id}^{+} = \frac{\langle W \rangle}{\langle W \rangle + \langle w_b \rangle} \tag{6}$$

$$\omega_{id}^{-} = \left(1 - \frac{1}{P}\right) \frac{W_0}{W_0 + \langle w_b \rangle} + \frac{1}{P} \tag{7}$$

where $W_0 = \langle W \rangle - \langle w_b \rangle / P$. Here, ω_{id}^{-} is constructed from the consideration that all cores but one have the workload W_0 and the remaining one has load $W_0 + \langle w_b \rangle$.

This analysis has identified that the ALL method is slightly superior to red the native HemeLB implementation. However, the performance of both methods is curtailed somewhat, not by poor load distribution but by individual cores that may take some 10-20% longer to process information. Identification of the reason for this is currently being investigated and will be published once a more detailed understanding of this performance issue red has been achieved.

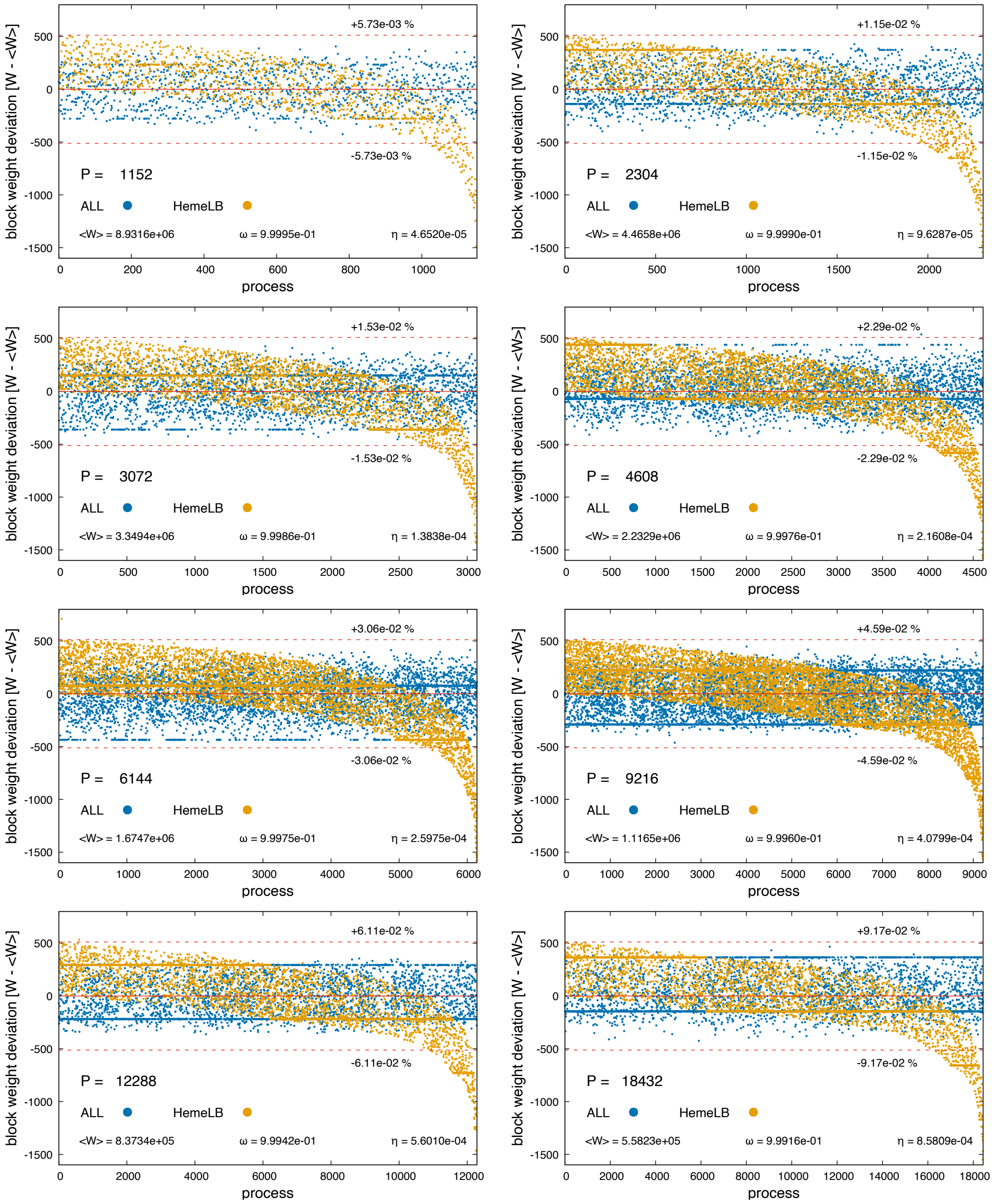

Figure 1: Comparison between the original HemeLB load balancing scheme and the histogram method from ALL for core counts $P \in [1152, 18432]$. The deviation of the total block weights, W_i, on each process from the average, $\langle W \rangle$ is shown. Solid red line indicates zero, while dashed red lines show the distance to the average block weight, $\langle w_b \rangle = 512$. Additionally, values for the performance measures, ω and η are given.

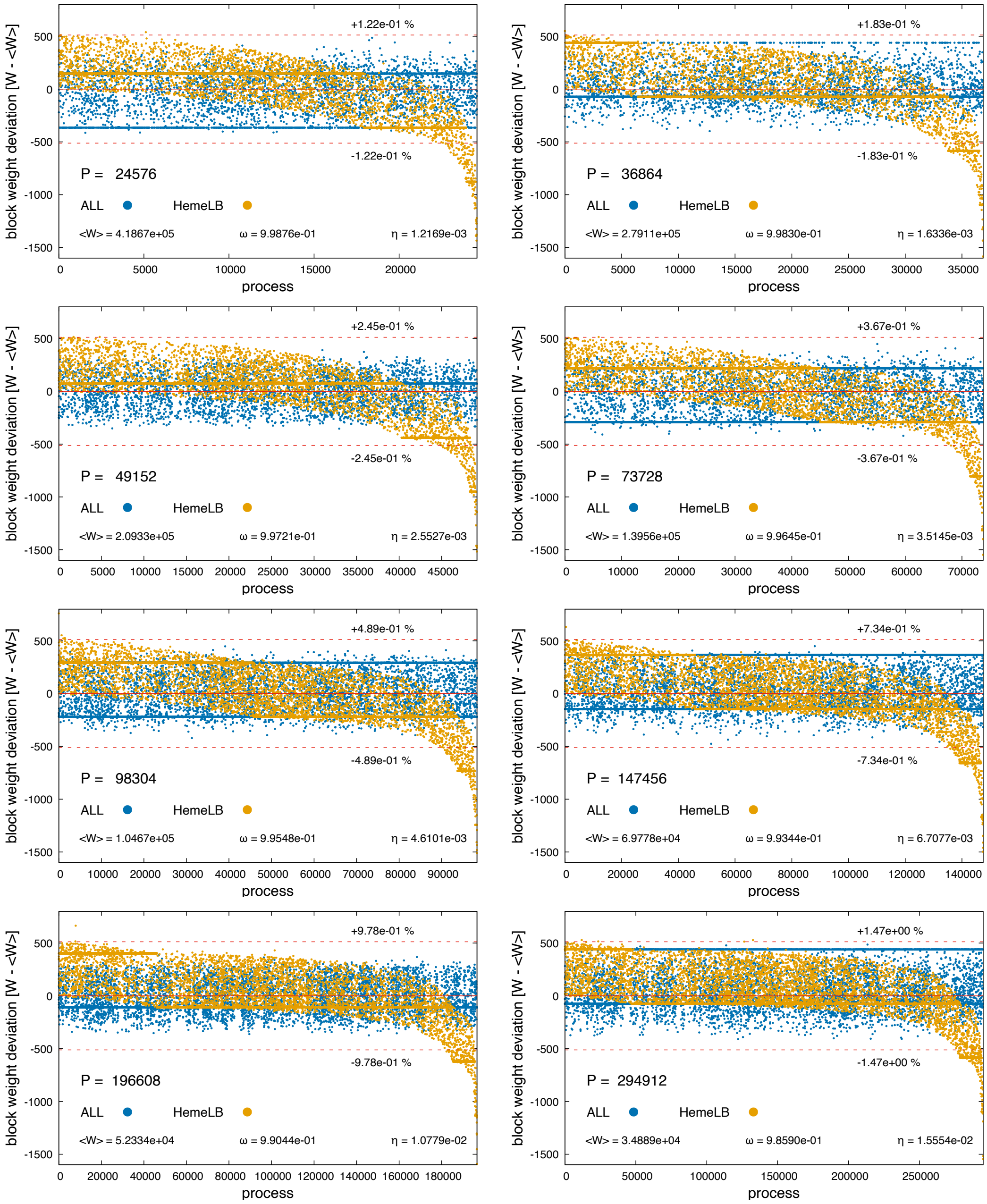

Figure 2: Same as in Fig. 1 for core counts $P \in [24576, 294912]$

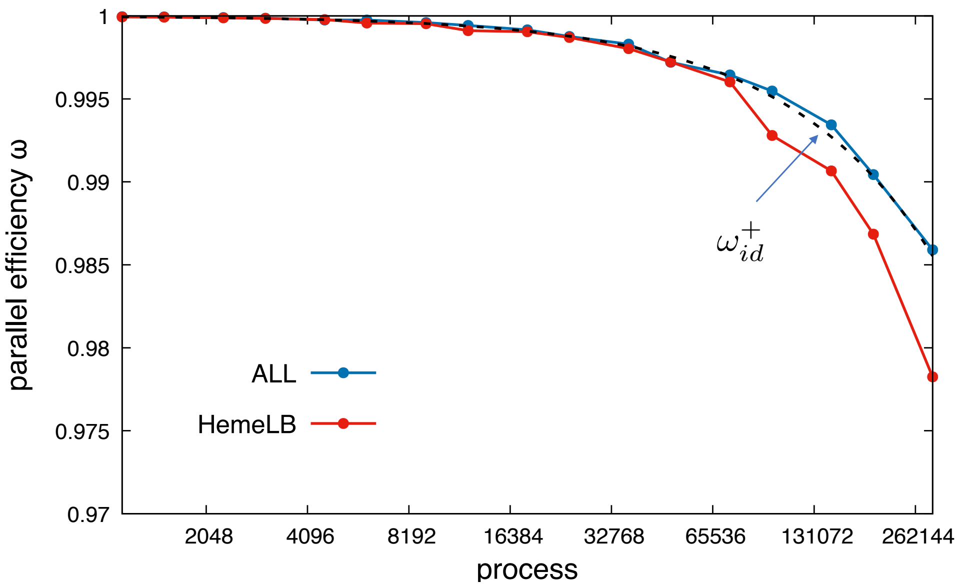

Figure 3: Parallel efficiency ω (Eq. 3) as function of core count. Results for HemeLB and the ALL library are compared. As a reference, the expected maximum deviation for an ideal distribution is shown as ω_{id}^{+} (cmp. text).

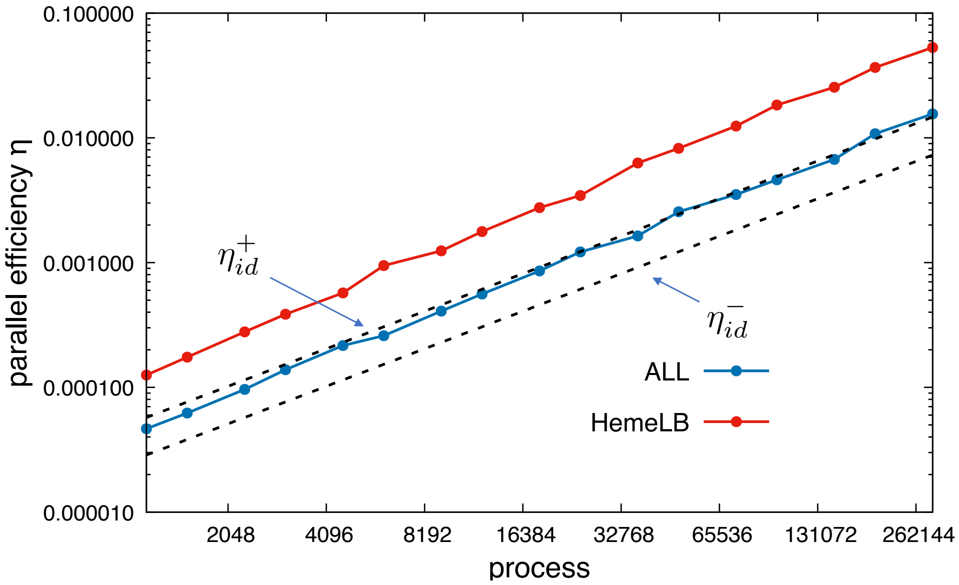

Figure 4: Parallel efficiency η (Eq. 4) as function of core count. Results for HemeLB and the ALL library are compared. As a reference, the expected maximum deviations for an ideal distribution are shown as $\eta_{id}^{\pm}$ (cmp. text).
